# Beneficial effect of continuous positive airway pressure on lipid profiles in obstructive sleep apnea: a meta-analysis

**DOI:** 10.1007/s11325-014-1082-x

**Published:** 2014-11-25

**Authors:** Ming-Tzer Lin, Hsien-Ho Lin, Pei-Lin Lee, Pei-Hsuan Weng, Chang-Chun Lee, Ting-Chun Lai, Wei Liu, Chi-Ling Chen

**Affiliations:** 1 Department of Internal Medicine, Hsiao Chung-Cheng Hospital, No. 15-1, Sec. 1, Nanya South Road, Banciao District, New Taipei, 220 Taiwan; 2 Graduate Institute of Epidemiology and Preventive Medicine, College of Public Health, National Taiwan University, 5Fl, No. 17, Hsu Chow Road, Taipei, 100 Taiwan; 3 Center of Sleep Disorder, National Taiwan University Hospital, No. 7, Zhongshan South Road, Taipei, 100 Taiwan; 4 Department of Internal Medicine, National Taiwan University Hospital, No. 7, Zhongshan South Road, Taipei, 100 Taiwan; 5 Department of Family Medicine, Taiwan Adventist Hospital, No. 424, Sec. 2, Bade Road, Songshan District, Taipei, 105 Taiwan; 6 Graduate Institute of Clinical Medicine, College of Medicine, National Taiwan University, No. 7, Zhongshan South Road, Taipei, 100 Taiwan

**Keywords:** Cholesterol, Continuous positive airway pressure, Lipoproteins, Meta-analysis, Sleep apnea, obstructive, Triglycerides

## Abstract

**Purpose:**

Dyslipidemia is considered as one mechanism causing cardiovascular sequelae in obstructive sleep apnea (OSA). Continuous positive airway pressure (CPAP) can reduce cardiovascular morbidities but its effect on lipid profiles is inconclusive. This study aimed to investigate the effects of CPAP on lipid profiles by a meta-analysis of the existing randomized controlled trials.

**Methods:**

Studies were retrieved from MEDLINE/PubMed, EMBASE, CENTRAL, commercial websites, and article references up to August 2013 following the protocols (PROSPERO CRD42012002636). Randomized controlled trials investigating the CPAP effects on changes in lipid profiles in adult patients with OSA were included. Two independent researchers extracted relevant data in duplicate. The pooled effect was analyzed by fixed-effect generic inverse variance, and the heterogeneity was assessed using the *I* ^2^ statistic.

**Results:**

Six trials with 348 patients and 351 controls were included. CPAP significantly lowered total cholesterol (mean, −6.23 mg/dl; 95% CI, −8.73 to –3.73; *I* ^2^, 0 %; *p* < 0.001), triglyceride (mean, −12.60 mg/dl; 95% CI, −18.80 to −6.41; *I* ^2^, 25 %; *p* < 0.001), and high-density lipoprotein (mean, −1.05 mg/dl; 95% CI, −1.69 to −0.40; *I* ^2^, 0 %; *p* = 0.001), but not low-density lipoprotein (mean, −1.01 mg/dl; 95% CI, −5.04 to 3.02; *I* ^2^, 0 %; *p* = 0.62). The lipid-lowering effects were homogeneous across the studies. By subgroup analysis, the reductions of lipid profiles were associated with the cross-over design, subtherapeutic CPAP as placebo, enrolled patients with moderate-to-severe OSA or daytime sleepiness, and CPAP treatment with short-term duration or good compliance.

**Conclusions:**

This meta-analysis validates the observation that CPAP can reduce lipid profiles in patients with OSA.

**Electronic supplementary material:**

The online version of this article (doi:10.1007/s11325-014-1082-x) contains supplementary material, which is available to authorized users.

## Introduction

Obstructive sleep apnea (OSA) is common in adults, with an estimated prevalence of 6–24 % [1]. It is characterized by recurrent collapse of the upper airway during sleep that can lead to chronic intermittent hypoxia and sleep fragmentation [2]. Evidences demonstrate that patients with OSA suffer from higher cardiovascular morbidities and mortalities [3].

Dyslipidemia, a known risk factor of atherosclerotic cardiovascular disease [4], has been posited to be responsible for the cardiovascular sequelae in OSA [5–7]. The chronic intermittent hypoxia in OSA can result in dyslipidemia through the upregulation of lipid biosynthesis, promotion of peripheral lipolysis, and suppression of lipoprotein clearance [8, 9]. Continuous positive airway pressure (CPAP) can reduce the risk of cardiovascular sequelae [3], but the exact mechanisms have not been fully elucidated where the reversal of dyslipidemia may be a crucial mechanism [8–11].

Studies evaluating the effects of CPAP on lipid metabolism had conflicting results. Most are observational and small-sized and without proper controls [12]. Although several randomized controlled trials (RCTs) have overcome such shortcomings, the results remain inconclusive. The discrepancies between these RCTs are related to the heterogeneity of participants, duration of CPAP treatment, placebo selections, small sample size, and lipid profile not set as the primary outcome [12]. To date, there have been two studies using meta-analysis to approach this issue. Robinson’s study composed of only two RCTs that meta-analyzed the pooled effects of CPAP on lipid profiles published one decade ago [13]. Another, Xu’s study, which was registered after ours (PROSPERO No. CRD42013005732) and published recently, included six RCTs for meta-analysis [14]. However, this study improperly included Kohler’s study which used CPAP withdrawal design [15] and Sharma’s study which was retracted [16]. It could lead to questionable results and conclusion. In addition, the study’s design and control type were not investigated of their confounding effects. Therefore, we reported our study here by meta-analysis of RCTs to investigate the effects of CPAP on lipid profiles up to August 2013 (PROSPERO No. CRD42012002636).

## Materials and methods

### Data extraction

The data extraction was conducted in duplicate by two independent researchers. When the results were inconsistent, a third person was invited and the decision was made by the majority.

**Fig. 1:**
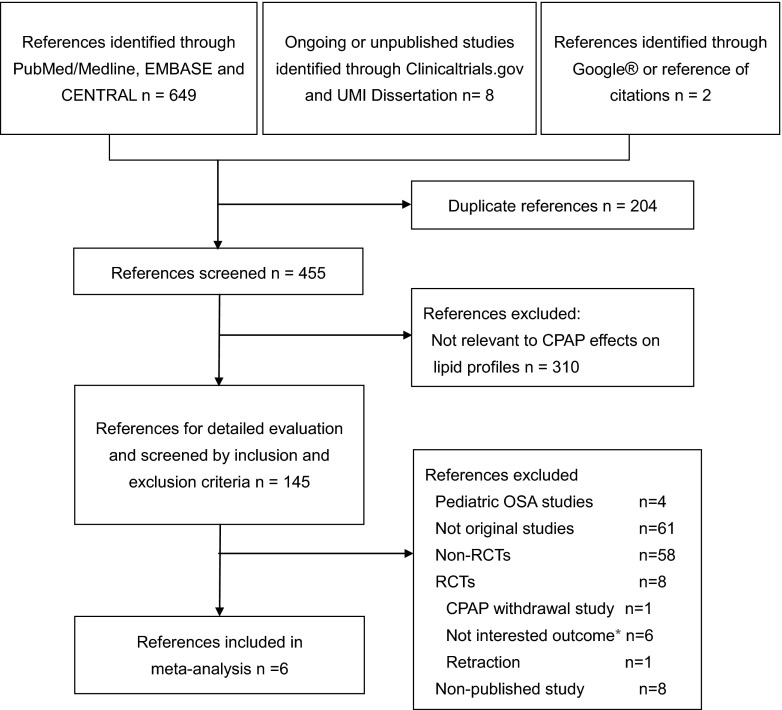
Flow diagram of the study. *RCTs* randomized controlled trials, *CPAP* continuous positive airway pressure, *OSA* obstructive sleep apnea. *Asterisk* indicates one study included without extractable outcome

The flow chart of data extraction (Fig. 1) showed that a web-based search was first conducted in the bibliographic databases of PubMed/MEDLINE, EMBASE, and the Cochrane Central Register of Controlled Trials (CENTRAL) updated to August 2013 using following strategies: (1) MeSH term approach: (“sleep apnoea, obstructive” [MeSH]) AND (“continuous positive airway pressure” [MeSH]) AND (“metabolic diseases” [MeSH] OR lipids [MeSH] OR cholesterol [MeSH] OR triglycerides [MeSH] OR lipoproteins [MeSH]); (2) direct keyword approach: (“obstructive sleep apnoea” OR OSA OR “obstructive sleep apnoea syndrome” OR OSAS) AND (“continuous positive airway pressure” OR CPAP) AND (dyslipidemia OR lipid(s) OR triglyceride(s) OR lipoprotein(s) OR LDL OR HDL OR TG OR “metabolic disease” OR cholesterol(s)). No limitation was set.

The ongoing or unpublished studies were accessed via the international trials register (Clinicaltrials.gov/) and the UMI Dissertation (ProQuest Dissertation and Theses Database). A commercial Internet search engine (Google®) was also searched using the same strategy. Additional publications were excluded from identified references.

Second, the titles and abstracts of identified studies were screened for potentially pertinent (containing any direct or indirect relevance to CPAP effect on lipid profiles), which were then retrieved from the full text. Studies were considered eligible for meta-analysis if they fulfilled all of the following criteria: (1) subjects were age ≥18 years old; (2) the study participants were diagnosed as OSA by polysomnography where apnea-hypopnea index (AHI), respiratory disturbance index (RDI), or oxygen desaturation index (ODI) was >5/h; (3) the studies were designed as RCT trials; (4) CPAP was the intervention; (5) lipid profiles, including total cholesterol (T-CHO), triglyceride (TG), high-density lipoprotein (HDL), and low-density lipoprotein (LDL), were the outcomes of CPAP treatment. Unpublished studies or studies without extractable outcome were excluded.

### Coding

The full texts of included papers were thoroughly reviewed, and the parameters were retrieved following the protocols (http://www.crd.york.ac.uk/PROSPERO/display_record.asp?ID=CRD42012002636). The information extracted included parameters on (1) study conditions, including sample size, source of participants, study design, and end points; (2) patient characteristics, including race, age, sex, body mass index (BMI), comorbidity, maintenance medications, OSA severity, daytime sleepiness, and use or non-use of lipid-lowering agents; (3) CPAP interventions including duration, categorized as long term (≥12 weeks) or short term (<12 weeks), and compliance, with average use ≥4 h/night as good compliance [17]; (4) levels of T-CHO, TG, HDL, and LDL at baseline and at the end of the trials; and (5) BMI and blood pressure (BP) before and after CPAP treatments.

The severity of OSA was categorized as moderate to severe if AHI or RDI was ≥15/h or ODI was >10/h [18]. Daytime sleepiness was assessed using the Epworth Sleepiness Scale (ESS), where sleepiness was defined as ESS ≥10.

### Risk of bias assessment

The risk of bias of the included studies was assessed from six domains: sequence generation, allocation concealment, blinding, incomplete outcome data, selective outcome reporting, and other issues [19].

### Statistical analysis

The software used for analysis included STATA software 9 (STATA Corporation, College Station, TX) and Review Manager (RevMan) 5.1.6 (Nordic Cochrane Centre, Copenhagen, Denmark). The serum levels of T-CHO, TG, HDL, and LDL were presented as mean ± standard deviation (SD) with 95 % confidence interval (95% CI) in milligrams per deciliter. The effective size was mean differences in changes of T-CHO, TG, HDL, and LDL between the CPAP and control groups. The variance of mean differences was calculated as Elbourne’s study [20].

The statistical heterogeneity was assessed using the *I*-square statistic (significance was set at *I*-square >75 %). The Dersimonian and Laird random effect models were used to evaluate differences in changes. Sensitivity analysis was applied to assess the robustness of the studies’ results when information was inadequate. The contour-enhanced plot with the *trim and fill* adjustments was used to assess publication bias [21].

Subgroup analysis and meta-regression were used to evaluate possible sources of bias, including study design (cross-over and control selection), age, sex, BMI, comorbidity, medications, OSA severity, daytime sleepiness, CPAP duration and compliance, baseline levels of T-CHO, TG, HDL, and LDL, and change in BP before and after CPAP treatment.

## Results

### Search results

Data extraction identified 145 potentially relevant studies (Fig. 1), and six RCTs with a total of 348 patients and 351 controls were finally enrolled after filtering with the inclusion and exclusion criteria.

### Trial characteristics

The characteristics of the six trials were summarized (Table 1). The sample number was <50 in four trials [22–25], and all of the participants were recruited from hospitals. Three studies were cross-over and three were parallel [13, 24, 26]. Three trials used therapeutic CPAP as placebo [13, 22, 25], while others used no treatment as controls.

**Table 1:** Characteristics of randomized controlled trials (*n* = 6) of continuous positive airway pressure (CPAP) effects on lipid profiles in obstructive sleep apnea

Author, year (country)	Number of case/control	Control type	Dropouts case/control (overall %)	Anti-lipid medications	AHI/RD/ODI (/h)	Age (years)	Male (%)	BMI (kg/m^2^)	DM	ESS	CPAP duration (weeks)	CPAP compliance (h/night) (SD or range or IQR)	Change of BP
Comondore 2009 (Canada) [23]	13	NT	0/0 (0)	NM	AHI >15, 27.9 (NM)	55.5 (7.1)	69	31.1 (NM)	NM	6.8 (NM)	4	5.5 (NM)	No
Coughlin 2007 (UK) [25]	35	SC	1/0 (2.9)	No	RDI >15, 39.7 (13.8)	49 (8.3)	100	36.1 (7.6)	No	13.8 (4.9)	6	3.9 (range, 0–7.4)	Reduced
Craig 2012 (UK, Canada) [26]	195/196	NT	41/40 (20.7)	Allowed	ODI >7.5, 9.8 (NM)	57.7 (7.3)	78	32.4 (5.6)	Yes	8.0 (4.3)	24	2.7^a^ (IQR, 0.6–5.0)	Elevated
Drager 2007 (Brazil) [24]	12/12	NT	0/0 (0)	No	AHI >30, 59 (21.7)	45.5 (6.6)	100	29.8 (2.9)	No	13.5 (4.5)	16	6 (range, 5–6.6)	Reduced
Phillips 2011 (Australia) [22]	37	SC	5/3 (21.6)	Allowed, except fibrate	AHI >25, 41.2 (23.9)	49 (13)	92	32.1 (4.3)	Yes	11.2 (4.9)	8	4.4 (2.2)	NM
Robinson 2004 (UK) [13]	113/112	SC	5/0 (2.2)	Allowed	ODI >10, 38.7 (20.7)	49.4 (10.3)	100	35.8 (7.0)	Yes	16.2 (3.3)	4	5 (1.9)	NM

The value was expressed as mean (standard deviation)

*NM* not mentioned, *AHI* apnea-hypopnea index, *RDI* respiratory disturbance index, *ODI* oxygen desaturation index, *ESS* Epworth Sleepiness Scale, *BMI* body mass index, *DM* diabetic mellitus, *HTN* hypertension, *CVD* cardiovascular disease, *NT* no treatment, *SC* subtherapeutic CPAP, *IQR* inter-quartile range

^a^Median value

The specifications for enrolment in the six trials included recruitment of patients with minimal symptoms in two [26, 23], free of comorbidities in one [24], no medications in two [25, 24], and exclusion of patients with substantial hypersomnolence in one [23]. In general, the participants were obese, middle-aged men with daytime sleepiness, and had comorbidities like hypertension (51 %) and diabetes (9 %). All except one trial included subjects with moderate-to-severe OSA [26]. Race was not specified in all of the trials.

The duration of CPAP treatment varied from weeks to months, including two long-term [26, 24] and four short-term. Compliance to CPAP was good in five and poor in two [25, 26].

Four studies addressed changes in BMI, where BMI decreased in one [26] and did not change in three [25, 24, 22]. Changes in blood pressure were reported in four trials, in which blood pressure decreased in two [25, 24], did not change in one [23], and increased in one [26].

### Risk of bias assessment

**Fig. 2:**
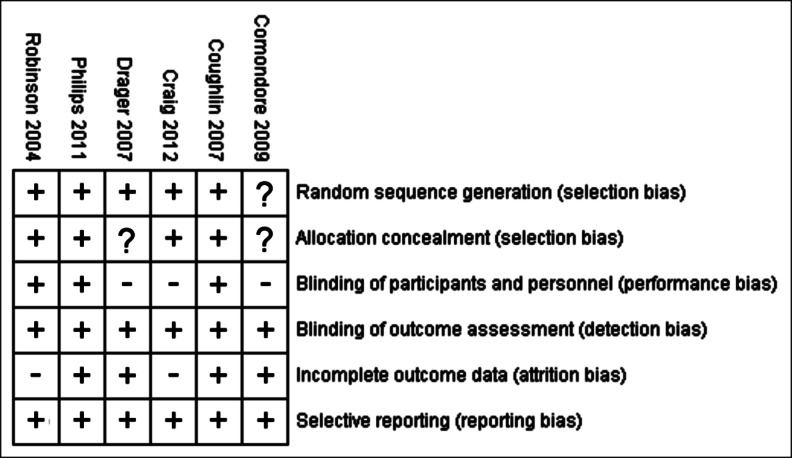
Summary figure of risk of bias. *Plus mark* (+) low risk of bias, *minus mark* (−) high risk of bias, *question mark* (?) unclear risk of bias

The heterogeneities of the enrolled studies (Fig. 2; Supplemental S1) revealed that five studies [25, 26, 5, 22, 13] properly randomized their participants and one did not mention details of the randomization [23]. Four studies reported the allocation procedure and blindness [13, 25, 26, 22]. Performance bias was marked as high risk in three studies [23, 26, 24] that did not use subtherapeutic CPAP as control. Attrition bias was noted in two studies reporting >20 % dropout rate [26, 22] and in one study with >50 % missing data without explanation [13].

### Main findings

**Fig. 3:**
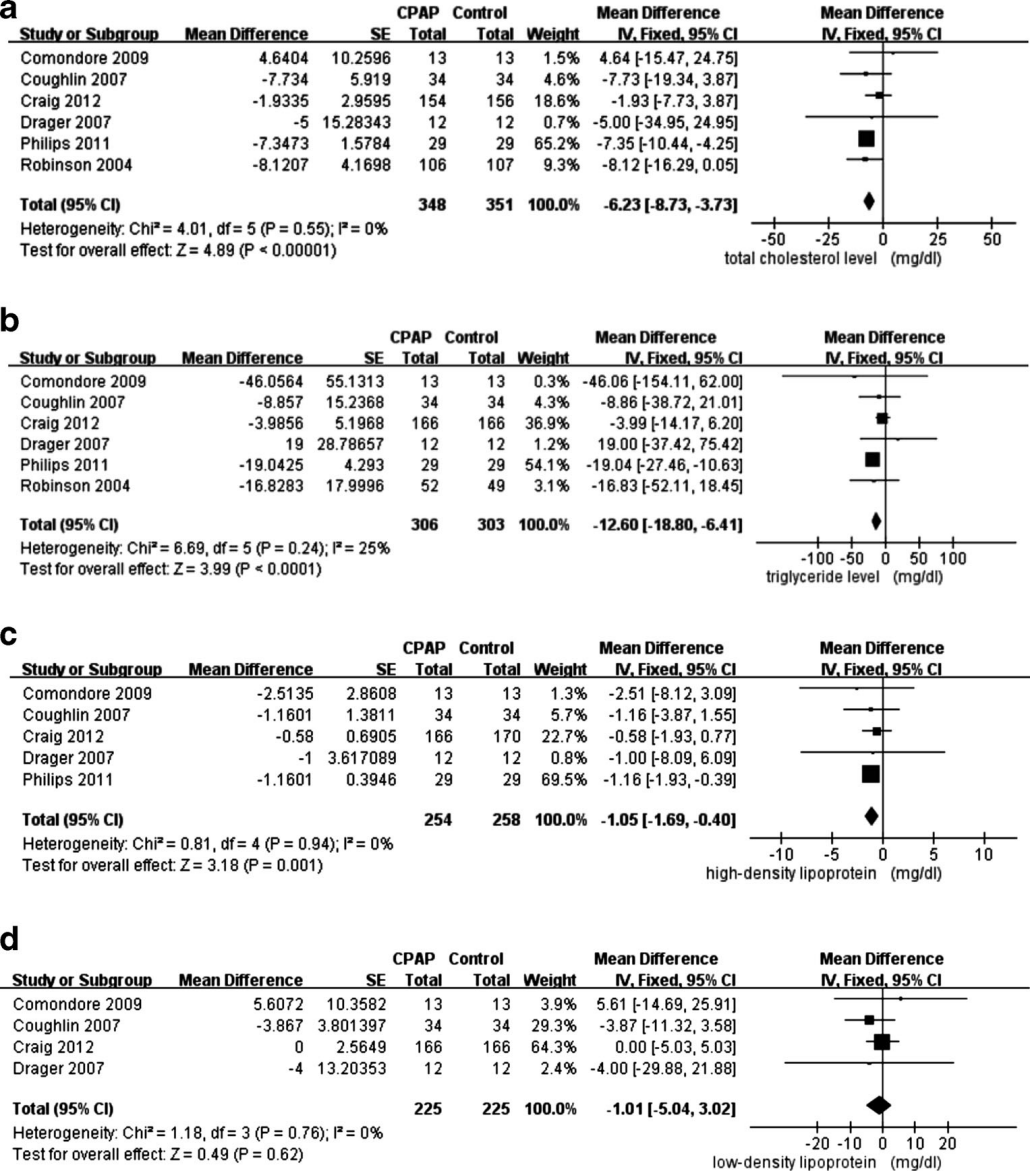
Fixed-effect meta-analysis of continuous positive airway pressure (*CPAP*) effects on lipid profiles in obstructive sleep apnea (*OSA*). **a** Total cholesterol. **b** Triglyceride. **c** High-density lipoprotein. **d** Low-density lipoprotein

The changes in T-CHO, TG, HDL, and LDL are shown in Table 2. The heterogeneity among studies analyzing T-CHO (*I* ^2^ 0 %), TG (*I* ^2^ 25 %), HDL (*I* ^2^ 0 %), and LDL (*I* ^2^ 0 %) were all low (Fig. 3). After applying the fixed model to determine the difference in changes between the CPAP treatment and control groups, the overall reduction was significant in T-CHO (mean change −6.23 mg/dl, 95% CI −8.73 to −3.73 mg/dl, *p* < 0.001) (Fig. 3a), TG (mean −12.60 mg/dl, 95% CI −18.80 to −6.41 mg/dl, *p* < 0.001) (Fig. 3b), and HDL level (mean −1.05 mg/dl, 95% CI −1.69 to −0.40 mg/dl, *p* = 0.001) (Fig. 3c). The LDL level was not affected by CPAP (mean −1.01 mg/dl, 95% CI −5.04 to 3.02, *p* = 0.62) (Fig. 3d).

**Table 2:** Baseline and outcome data of lipid profiles of randomized controlled trials (*n* = 6) on continuous positive airway pressure effects on lipid metabolism in obstructive sleep apnea

Studies	Baseline, level (mg/dl) (SD)				Effect size (mg/dl) (95 % CI)			
	T-CHO	TG	HDL	LDL	T-CHO	TG	HDL	LDL
Comondore 2009 [23]	185.62 (NM)	162.08 (NM)	44.47 (NM)	109.82 (NM)	4.64 (−15.47~24.75)	−46.06 (−154.11~62)	−2.51 (−8.12~3.09)	5.41 (−14.69~25.91)
Coughlin 2007 [25]	NM (NM)	NM (NM)	NM (NM)	NM (NM)	−7.73 (−19.34~3.87)	−8.86 (−38.72~21.01)	−1.16 (−3.87~1.55)	−3.87 (−11.32~3.58)
Craig 2012 [26]	203 (44.47)	149.24 (84.1)	50.27 (13.8)	121.23 (38.46)	−1.93 (−7.73~3.87)	−3.99 (−14.17~6.2)	−0.58 (−1.93~0.77)	0 (−5.03~5.03)
Drager 2007 [24]	234 (38.25)	161.5 (56.73)	49.5 (8.99)	152.5 (34.08)	−5 (−34.95~24.95)	19 (−37.42~75.42)	−1 (−8.09~6.09)	−4 (−29.88~21.88)
Phillips 2011 [22]	197.60 (39.83)	170.05 (86.80)	45.24 (10.83)	121.04 (38.28)	−7.35 (−10.44~−4.25)	−19.04 (−27.46~−10.63)	−1.16 (−1.93~−0.39)	NM
Robinson 2004 [13]	218.49 (46.48)	262.2 (198.92)	NM	NM	−8.12 (−16.29~0.05)	−16.83 (−52.11~18.45)	NM	NM

The value was expressed as mean

*NM* not mentioned, *SD* standard deviation, *T-CHO* total cholesterol, *TG* triglyceride, *HDL* high-density lipoprotein, *LDL* low-density lipoprotein

The subgroup analysis (Supplemental S2) revealed that CPAP lowered T-CHO, TG, and HDL in studies with cross-over design, subtherapeutic CPAP as placebo, moderate-to-severe OSA, daytime sleepiness, short-term CPAP treatment, use of lipid-lowering agents, good compliance to CPAP, and low risk of bias (Supplemental Figs. S2a–c). This was not seen in LDL (Supplemental Fig. S2d).

Meta-regression showed that the TG-lowering effect increased in studies with shorter CPAP duration (*β* = 0.87, *p* = 0.029) and with more patients with hypertension (*β* = 0.23, *p* = 0.049) and cardiovascular disease (*β* = 2.30, *p* = 0.040) (Supplemental Table S4A).

There was no major publication bias observed (Supplemental S3).

### Sensitivity analysis

After excluding Phillip’s study [22], which weighed more than 50 %, the CPAP effect on HDL (mean −0.78 mg/dl, 95% CI −1.95 to 0.38 mg/dl, *p* = 0.19) became insignificant. After excluding three studies with performance bias [23, 26, 24] and two studies with attribution bias [26, 13], the CPAP effects on lipid profiles did not change. Excluding the studies reporting changes in BMI [23, 26, 13], the CPAP effect on all lipid profiles remained unchanged (Supplemental Table S4B).

## Discussion

This study aimed to clarify the effects of CPAP on lipid profiles in patients with OSA through the meta-analysis of the RCTs updated to August 2013. Six RCTs with 348 patients and 351 controls were analyzed. Most of the participants were middle-aged, obese males with moderate-to-severe OSA. The results demonstrated that CPAP therapy significantly lowered T-CHO, TG, and HDL levels where these effects were homogeneous among studies. The subgroup analysis showed that the reduction in lipid profiles was associated with a cross-over design, subtherapeutic CPAP as placebo, enrolment of patients with moderate-to-severe OSA or daytime sleepiness, and CPAP treatment with short-term duration or good compliance.

The evidence towards such conclusion is strong when assessing using the GRADE approach (Supplemental S5). Compared to Robinson’s meta-analysis [13], this study systemically reviewed updated evidence and studied the CPAP effects on HDL and LDL. Different from Xu’s study [14], our meta-analysis did not enroll Kohler’s study, which investigated the change of lipid profiles after withdrawal of CPAP [15]. We also excluded Sharma’s study [27], which was retracted due to transcription errors on the assessment of abdominal fat, although these errors did not affect the blood sampling of lipid profiles [16]. Even including Sharma’s study, the CPAP effect on lipid profiles remained unchanged (Supplemental Table S4B). Besides, we found another two studies [24, 22] which were relevant to this issue but not included in Xu’s study. Furthermore, the confounding effects of the study’s design or control type were investigated in our study by subgroup analysis.

A number of studies have investigated the effect of CPAP on lipid profiles, but the results were rather conflicting [12]. Majority of these studies were observational studies, which was easily confounded by unmeasured factors. For example, positive studies mostly revealed that CPAP could improve T-CHO and TG levels in OSA patients with weight reduction, good CPAP adherence, or initial abnormal lipid profile [17, 28–31]. Negative studies often had small sample size or lacked information of CPAP compliance [32–34]. In contrary, present study meta-analyzed the RCTs which could eliminate those confounders and drew the solid conclusion of the lipid-lowering effect of CPAP.

Our result showed that CPAP had only modest effect on T-CHO and TG, minimal on HDL, and no effect on LDL. In one recent study, the meta-regression analysis showed the AHI had significant effect for LDL and TG while age had significant effect for T-CHO, LDL and HDL, and BMI had significant effect for LDL and HDL [35]. Our study only recruited RCTs where the CPAP effect on lipid profiles was rather related on reversal of apnea-hypopnea than confounders like age and BMI. This may explain why CPAP had a greater impact on TG level than other lipid profiles in the present study. Moreover, our finding that HDL decreased after CPAP treatment echoed the findings in Cuhadaroglu’s study [31] but contradicted other observational studies [36, 28]. All of these studies, designed as before-after series, had no proper control. Previous studies also noted that OSA patients who adhered to CPAP were more compliant in refilling their lipid-lowering medications [37]. Thus, using before-after series studies to investigate CPAP effects would be biased towards the more beneficial effects on HDL. In the current meta-analysis, even after confounders originating from lifestyle were controlled in RCTs or after excluding an influential study with high weight [22], CPAP still had no benefits on HDL.

Although CPAP is beneficial for affecting lipid metabolism, the magnitude of lipid reduction is modest (3.0 % for T-CHO, 6.7 % for TG, 3.1 % for HDL, and 1.2 % for LDL) compared to the effect of anti-lipid agents (>30 %) [38]. To estimate the 10-year risk of developing coronary heart disease based on the Framingham study score system [39], such reduction in lipid profiles only represent about 1~2 % of vascular disease risk reduction. Lipid-lowering drugs are still needed to treat dyslipidemia in OSA. On the other hand, the subgroup analysis here shows that the lipid-lowering effects are also noted in studies where participants are allowed to use lipid-lowering agents, which may support the synergistic effect of CPAP and lipid-lowering agents like valsartan to reduce BP [40]. Further studies to elucidate whether CPAP interacts with lipid-lowering agents to enhance their effects are warranted.

The studies with cross-over design or those that use subtherapeutic CPAP as control show more prominent lipid-lowering effects. This is related to the less heterogeneity of participants in the cross-over design and minimal lifestyle-related confounders with subtherapeutic CPAP as control. The lipid-lowering effects are more significant in studies recruiting participants with moderate-to-severe OSA or daytime sleepiness. The former is associated with more episodes of intermittent hypoxia [41], one proposed mechanism causing dyslipidemia in OSA [8]. Hence, CPAP, by reverting intermittent hypoxia, can have greater impact on relieving dyslipidemia. The study of Barcelo et al. supports the view that daytime sleepiness is a useful marker for CPAP effect on cholesterol [42]. Moreover, the present meta-regression shows that the lipid-lowering effects are decreased in studies enrolling more patients with hypertension or cardiovascular disease. This may be because dyslipidemia in these patients can be induced by causes other than intermittent hypoxia, so the effects of CPAP on lipid profiles are compromised. The study of Kumor et al. reports the same phenomenon that CPAP decreases lipid concentration only in pure OSA, not in OSA with ischemic heart disease [43].

Several studies have proposed that CPAP compliance will impact the effects of CPAP on lipid metabolism [29, 44, 17]. This study confirms the observation that CPAP can lower lipid profiles in patients using CPAP more than 4 h per night. In the present study, the duration of CPAP treatment is inversely correlated to the CPAP effect on lipid profiles, which may be due to the influence of confounders other than CPAP, such as diet or exercise, which increases as the trial period becomes longer.

The risk of bias also influences the effects of CPAP on lipid profiles, especial performance bias. It may be related to the cross-over design or use of subtherapeutic CPAP, as previously mentioned.

The exact mechanism how CPAP improved lipid metabolism was not clear. The elimination of chronic intermittent hypoxia and sympathetic hyperactivity, two hallmarks of OSA, had been proposed as mechanisms. In animal model, chronic intermittent hypoxia had been demonstrated to promote dyslipidemia through upregulating the transcriptional factor, sterol regulatory element-binding protein-1, and downstream enzyme of triglyceride and phospholipid biosynthesis, stearoyl-CoA desaturase-1 [45]. Catecholamine could promote gluconeogenesis, peripheral insulin resistance, and lipolysis [46]. Therefore, by reversing the intermittent hypoxia and sympathetic hyperactivity, CPAP could improve dyslipidemia. In addition, by improving the hypersomnolence, CPAP could increase daytime physical activity and caloric expenditure which helped to improve dyslipidemia [47].

Previous studies suggested that intermediary mechanisms, like inflammation or sympathetic activation, may take part in linking intermittent hypoxia to dyslipidemia [46]. To explore the influence of inflammatory mediators and sympathetic hyperactivity on fat metabolism, we extracted parameters associated with inflammation or autonomic stress (Supplement S2, Table S2A-S2B) which included three studies regarding inflammatory mediators [23, 24, 13] and four regarding autonomic system [22–25]. Thereafter, we studied the effect size of lipid profiles through stratifying studies as containing inflammatory mediators or autonomic activity marker. The result showed that the CPAP lipid-lowering effect on T-CHO, TG, and HDL was only significant in studies where autonomic hyperactivity was also lowered by CPAP. The correlation between change in inflammatory parameters and the effect of CPAP on lipid profiles could not be concluded because only a few of recruited studies provided such information. However, one recent study by Joyeux-Faure showed that the statin therapy known for anti-inflammatory effect had no effect on inflammatory or autonomic activity markers despite its lipid-lowering effect [48].

This study has some limitations. First, the lipid profiles were not the primary outcomes in the majority of the studies included. Information on some important confounding factors like diet, physical activity, and body composition during the study period was not addressed [12]. Instead, changes of BMI were used as the surrogate of change in lifestyle during the trials. Even excluding studies reporting changes of BMI during the trial period, the effects of CPAP on lipid profiles remained unchanged. Secondly, all recruited study had CPAP duration less than 24 weeks, so the results could not be applied to those on CPAP more than 24 weeks. Lastly, difference in blood sampling timing (fasting or non-fasting) was noted between included studies, and this difference was equally distributed in the intervention and control groups because of the randomization procedure. Therefore, it should not bias our comparison.

## Conclusions

The independent benefits of CPAP on lipid profiles, including T-CHO and TG-lowering effects, are confirmed in this meta-analysis. The benefits are consistent among studies but are particularly prominent in patients with moderate-to-severe OSA, daytime sleepiness, good compliance to CPAP, or short-duration CPAP treatment. The cross-over design and use of subtherapeutic CPAP, which can influence the risk of bias, are factors affecting the effects of CPAP on lipid profiles. The benefit to HDL by CPAP is not proven. Due to the modest benefits of CPAP, lipid-lowering agents are still needed for better control of abnormal lipid levels in patients with OSA. Both CPAP and lipid-lowering agents may have synergistic effect, which warrants further studies.

## Electronic supplementary material

Below is the link to the electronic supplementary material.

ESM 1 (DOCX 3093 kb)
