# Supplementary material for: Beneficial effect of continuous positive airway pressure on lipid profiles in obstructive sleep apnea: a meta-analysis
Source: Sleep Breath. 2014 Nov 25;19(3):809–17. doi: 10.1007/s11325-014-1082-x (PMC4559086; doi:10.1007/s11325-014-1082-x)
Supplement: Supplementary file 1 — (DOCX 3093 kb) [file 11325_2014_1082_MOESM1_ESM.docx]

**Online Supplement**

**Continuous positive airway pressure is beneficial for lowering lipids in obstructive sleep apnoea: a meta-analysis**

Table of Contents

**SUPPLEMENT S1**

Study risk of bias assessment 2

**SUPPLEMENT S2**

Subgroup analysis 11

**SUPPLEMENT S3**

Analysis for publication bias 16

**SUPPLEMENT S4**

Sensitivity analysis 21

**SUPPLEMENT S5**

GRADE evidence profiles 25

**REFERENCES** 29

**Supplement S1.** Table on risk of bias for each included study

| **Study** | Comondore 2009 [[1](#_ENREF_1)] | Coughlin 2007 [[2](#_ENREF_2)] | Craig 2012 [[3](#_ENREF_3)] |
| --- | --- | --- | --- |
| **Bias** | **Authors' judgement**  **Support for judgement** | **Authors' judgement**  **Support for judgement** | **Authors' judgement**  **Support for judgement** |
| Random sequence generation (selection bias) | Unclear risk  Quote: “Subjects were then randomized to either CPAP or no therapy...”  Comment: no mention about random sequence generation | Low risk  Quote: “Randomisation used **a computer-generated sequence of random numbers**”  Comment: Probably done. | Low risk  Quote: “Randomisation was carried out by telephoning the Medical Research Council Clinical Trials Unit (MRC CTU), using **minimisation** with a random element of 80%; the minimisation factors were OSA severity (ODI, above or below 20/h), risk score (above or below 40) and participating centre.”  Comment: probably done. |
| Allocation concealment (selection bias) | Unclear risk  Quote: “Subjects were then randomized to either CPAP or no therapy...”  Comment: no mention about allocation concealment | Low risk  Quote: “Randomisation used a computer-generated sequence of random numbers and **CPAP was provided by a technician unconnected with the study**...”  Comment: Probably done. | Low risk  Quote: “Randomisation was carried out by **telephoning the Medical Research Council Clinical Trials Unit (MRC CTU)**…”  Comment: probably done. |
| Blinding of participants and personnel (performance bias) | High risk  No blindness was designed in this study. | Low risk  Quote: “Identical **sham CPAP** (Aria LX; Respironics Inc., Pittsburgh, PA, USA), modified as previously described”  Comment: it used sub-therapeutic CPAP as double-blinded design. Probably done. | High risk  Quote: “Sham CPAP was not used in the control arm and therefore **patients were not blinded**. **It was not possible to blind all trial staff**, although the assessments were done blind wherever possible. Therefore, the observed effects on sleepiness might be considered due to bias or the ‘**placebo effect**’ of CPAP.”  Comment: It possibly introduced this bias. |
| Blinding of outcome assessment (detection bias) | Low risk  Although intervention was not blinded, outcome was blood lab data, which was objective result. It was less likely to introduce this bias. | Low risk  It used sub-therapeutic CPAP as double blinded design, and outcomes was objective blood lab data. It was less likely to introduce this bias. | Low risk  Although intervention was not blinded, outcome was blood lab data, which was objective result. It was less likely to introduce bias. |
| Incomplete outcome data (attrition bias) | Low risk  No loss to follow-up was found from both groups. | Low risk  Only 1/35 (2.9%) missing from the active CPAP group for personal reasons.  Quote: “Data were analysed on an **intention-to-treat (ITT) basis**, including all data obtained even if patients were known not to be complying with CPAP therapy. ”  Comment: it was less likely to introduce bias. | High risk  Up to 20.7% of missing data was reported: for cholesterol, 41/195 missing from the active CPAP group and 40/196 missing from the sub-therapeutic CPAP group; for triglyceride, 27/195 missing from the active CPAP group and 25/196 missing from the sub-therapeutic CPAP group; for HDL, 25/195 missing from the active CPAP group and 27/196 missing from the sub-therapeutic CPAP group; for LDL, 29/195 missing from the active CPAP group and 30/196 missing from the sub-therapeutic CPAP group.  Quote: “Data were analysed on an **intention-to-treat basis** but excluded those with missing data…”; “**Forty-three (22%)** of the 195 patients randomised to CPAP reported stopping treatment during follow-up…”  Comment: Loss to follow up possibly related to outcome and was not by chance. It was likely to introduce this bias. |
| Selective reporting (reporting bias) | Low risk  No available protocol to evaluate reporting bias; however, all outcome described in Methods were reported. So it probably didn't introduce this bias. | Low risk  No available protocol to evaluate reporting bias; however, all outcome described in Methods were reported. So it probably didn't introduce this bias. | Low risk  It reported as protocol: ISRCTN34164388 and no selective reporting was found. |

| **Study** | Drager 2007 [[4](#_ENREF_4)] | Phillips 2011 [[5](#_ENREF_5)] | Robinson 2004 [[6](#_ENREF_6)] |
| --- | --- | --- | --- |
| **Bias** | **Authors' judgement**  **Support for judgement** | **Authors' judgement**  **Support for judgement** | **Authors' judgement**  **Support for judgement** |
| Random sequence generation (selection bias) | Low risk  Quote: “The participants were randomly assigned to no treatment (control) or treatment with CPAP ..., according to **a computer-generated list of random numbers**.”  Comment: probably done. | Low risk  Quote: “We used a **computer program** to produce the random treatment sequence using random block sizes of two, four, and six.”  Comment: probably done. | Low risk  Quote: “Patients were randomly assigned either therapeutic or sub-therapeutic NCPAP by use of a series of opaque sealed envelopes prepared in advance of the trial.”  Comment: probably done with random code sealed in envelopes. |
| Allocation concealment (selection bias) | Unclear risk  No description about allocation concealment was found in text. | Low risk  Quote: “These were stored in **sequentially numbered opaque envelopes**. The project manager was responsible for the allocation consignment and had no contact with any patient before or during the trial.”  Comment: probably done. | Low risk  Quote: “Patients were randomly assigned either therapeutic or sub-therapeutic NCPAP by use of a series of **opaque sealed envelopes** prepared in advance of the trial.”  Comment: probably done. |
| Blinding of participants and personnel (performance bias) | High risk  Quote: “**Patients were aware of their treatment assignments** and a placebo group using CPAP with ineffective pressure to open the airway was not included.” but “On the other hand, the key measurements of vascular outcome were obtained by one researcher blinded to treatment assignment. Furthermore, the control group had no significant changes in cardiovascular outcomes, results confirmed by the stability of the measurements over the 4-month study period.”  Comment: No blindness was found from study design, except observers. It still possibly introduced this bias. | Low risk  Quote: “The real and placebo CPAP devices (Remstar Auto; Philips Respironics, Murrysville, PA) used in this trial **are identical in appearance** and are currently being used in other large-scale randomized controlled trials”  Comment: Probably done. | Low risk  Quote: “Sub-therapeutic (control) NCPAP was **identical** to therapeutic NCPAP, except that pressure at the mask was unlikely to be enough to splint open the pharynx.” and “Patients were not aware of whether they were receiving therapeutic or sub-therapeutic nCPAP, and the nurse who assigned patients to treatment group did not take part in outcome assessments. The investigators who assessed outcome were unaware of the randomisation status of the patients, and did not set up or maintain the machines, or assist the patients. Therefore, despite the physical nature of the treatment, **the study was effectively double blind**.”  Comment: probably done. |
| Blinding of outcome assessment (detection bias) | Low risk  Quote: “The key measurements of vascular outcome were obtained by **one researcher blinded to treatment assignment**.”  Comment: Although intervention was not blinded, outcome was blood lab data, which was objective result. It was less likely to introduce bias. | Low risk  Quote: “All assays were performed by the biochemistry department at Royal Prince Alfred Hospital **using standard techniques**.” and “We attempted to blind outcomes assessors by limiting knowledge of treatment allocation to two people - the project manager and the trial physician - neither of whom interacted with the patients during the trial. The exception to this was when the trial physician was involved in withdrawing a patient. The project manager was responsible for configuring and monitoring compliance with all CPAP devices, including determining the therapeutic pressure at which to set the real CPAP.”  Comment: It used sub-therapeutic CPAP as double blinded design, and outcome was objective blood lab data. It was less likely to introduce this bias. | Low risk  Quote: “Sub-therapeutic (control) nCPAP was **identical** to therapeutic nCPAP, except that pressure at the mask was unlikely to be enough to splint open the pharynx.” and “Patients were not aware of whether they were receiving therapeutic or sub-therapeutic nCPAP, and the nurse who assigned patients to treatment group did not take part in outcome assessments. The investigators who assessed outcome were unaware of the randomisation status of the patients, and did not set up or maintain the machines, or assist the patients. Therefore, despite the physical nature of the treatment, **the study was effectively double blind**.”  Comment: probably done. |
| Incomplete outcome data (attrition bias) | Low risk  No loss to follow-up was found from both groups. | Low risk  There were 21.6% lost to follow-up, 5/37 missing from the active CPAP group; and 3/37 missing from the sub-therapeutic CPAP group. Reasons were the same between patients who completed the study or dropped out.  Quote: “We also performed an **intention-to-treat analysis** on the primary outcome.”  Comment: Loss to follow up did not relate to outcome and was by chance. It was less likely to introduce this bias. | High risk  2.2 % lost to follow-up but up to 52.8% of missing data was reported: for cholesterol, 5/112 missing from the sub-therapeutic CPAP group and 2/108 missing from the active CPAP group; for triglyceride, 63/112 missing from the sub-therapeutic CPAP group and 56/108 from active CPAP group.  No reasons were reported for those missing. It possibly introduced bias. |
| Selective reporting (reporting bias) | Low risk  It reported as protocol: NCT00400543 and no selective reporting was found. | Low risk  It reported as protocol: ACTRN 12605000066684 (www.anzctr.org.au) and no selective reporting was found. | Low risk  No available protocol to evaluate reporting bias; however, all outcome described in Methods were reported. So it probably didn't introduce this bias. |

**Supplement S2.** Sub-group analysis for total cholesterol (T-CHO), triglyceride (TG), high-density lipoprotein (HDL), and low-density lipoprotein (LDL). RCT, randomised controlled trials; OSA, obstructive sleep apnoea; CPAP, continuous positive airway pressure; BP, blood pressure; The *p* value was from the test statistic for testing heterogeneity between sub-groups. Forest plots for subgroup analysis of CPAP effects on **(S2A)** T-CHO, **(S2B)** TG, **(S2C)** HDL, and **(S2D)** LDL. Sub-group analysis by inflammatory and autonomic markers was provided (**Table S2A and S2B**).

**Figure S2A**

**
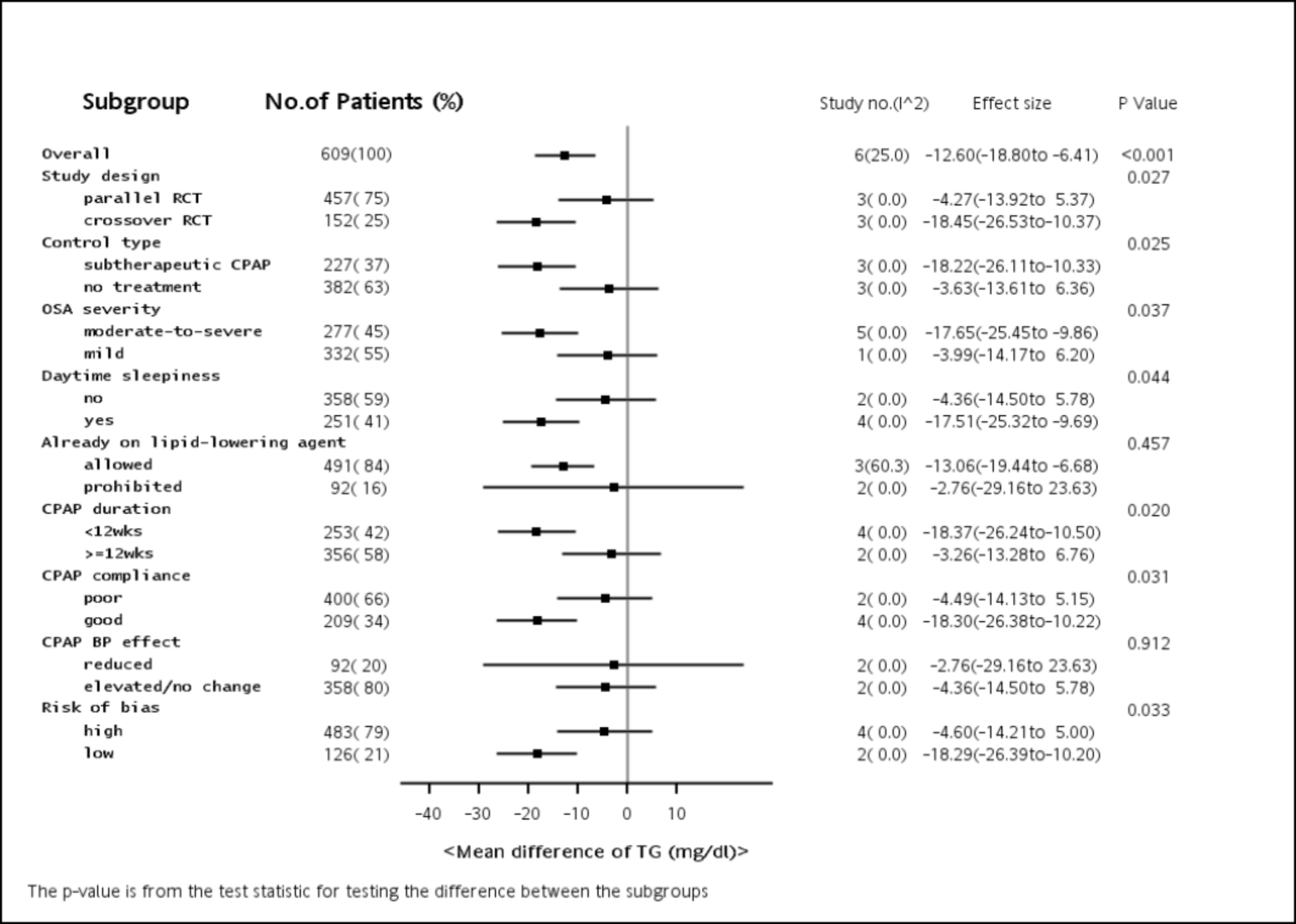
**

**Figure S2B**

**
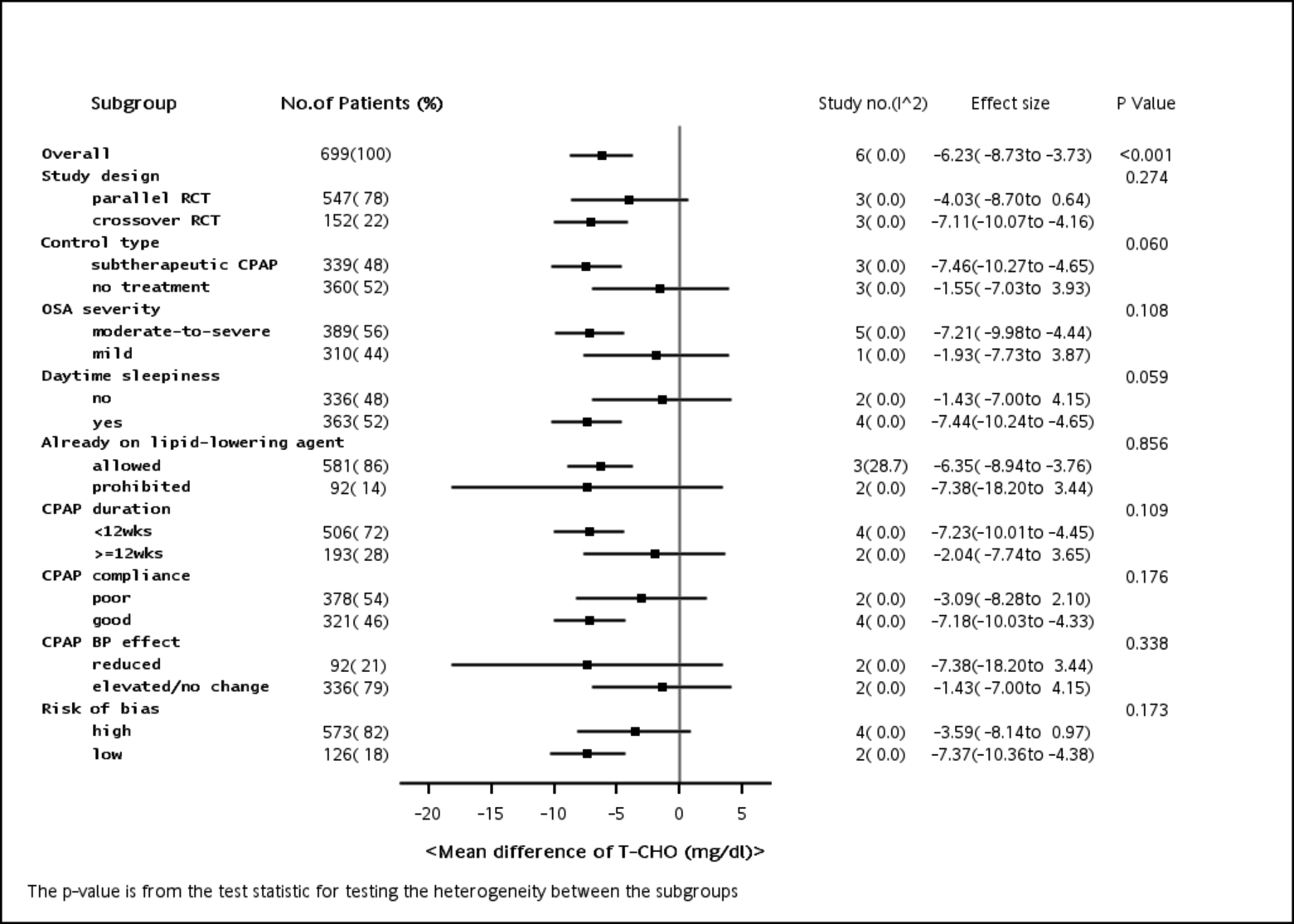
**

**Figure S2C**

**
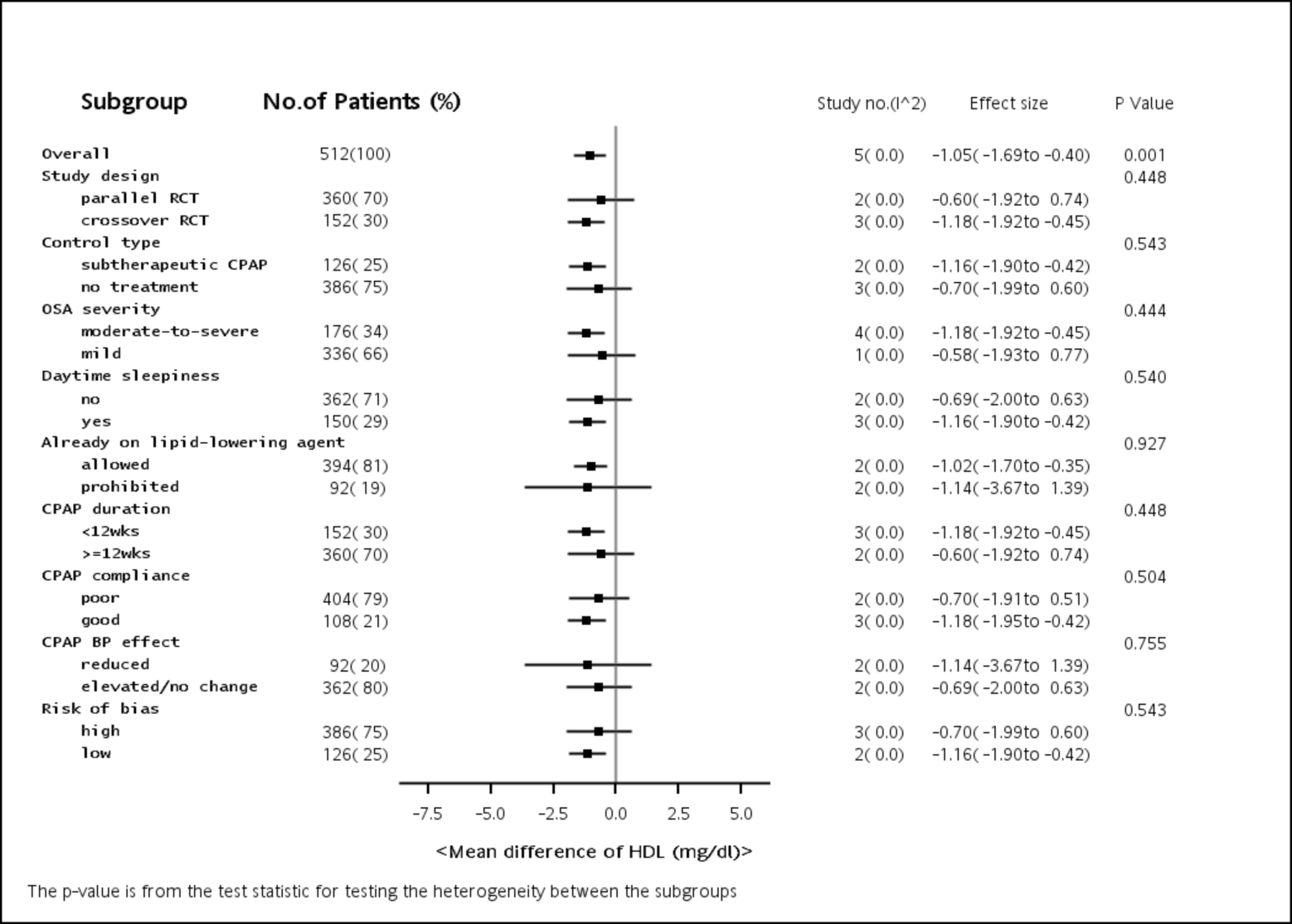
**

**Figure S2D**

**
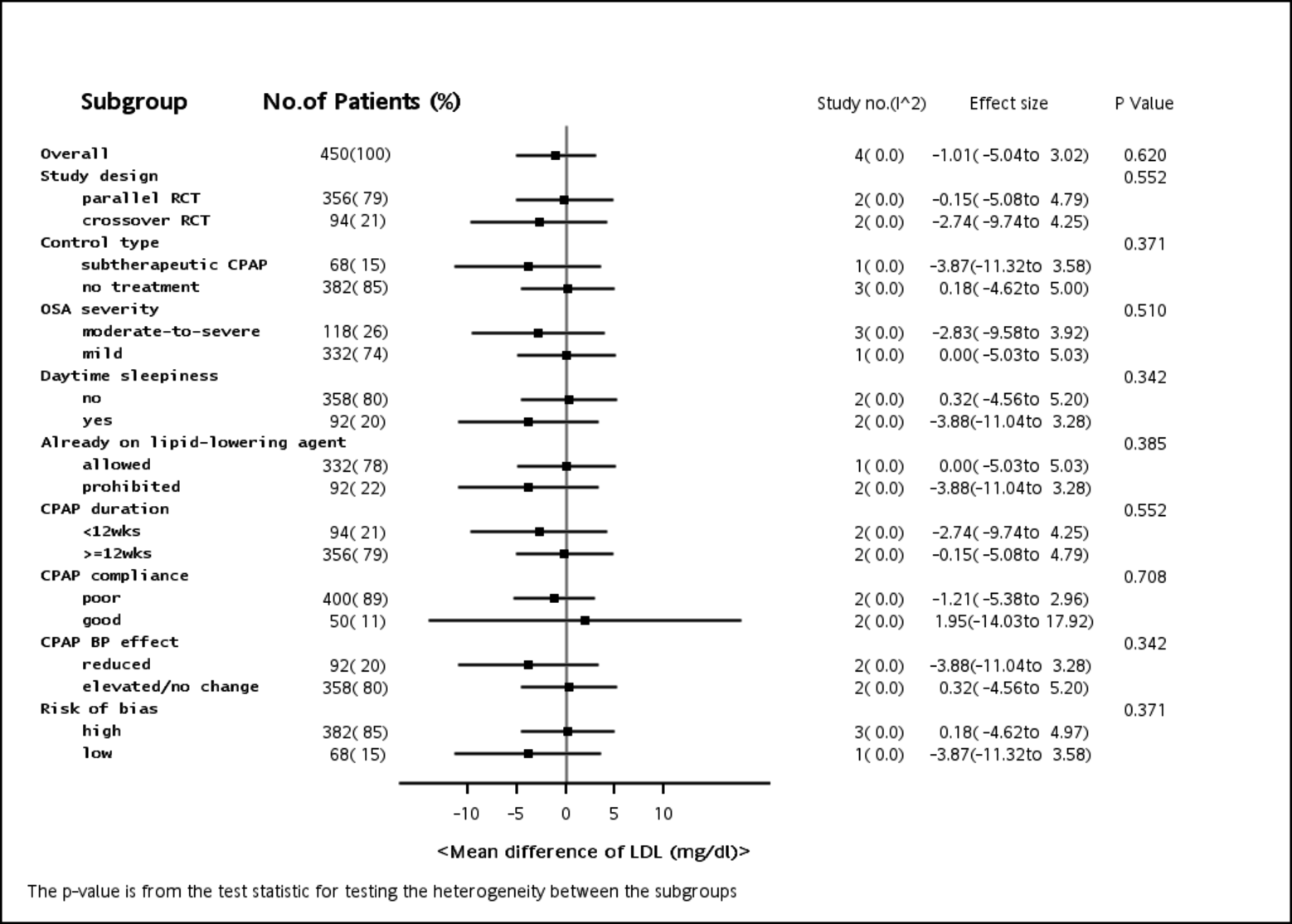
**

**Table S2A. Inflammatory mediator or autonomic activity markers extracted from the recruited studies.**

|  | **Inflammatory mediator** | **Effect size, mean (95%/CI)** | **P value** | **Autonomic activity marker** | **Effect size, mean (95%/CI)** | **P value** |
| --- | --- | --- | --- | --- | --- | --- |
| Comondore 2009 | CRP | 1.4 (-4.97, 7.77) | p=0.75 | Urine norepinephrine-to-creatinine ratio | -2.65 (-11.92, 6.61) | p=0.76 |
|  | Homocysteine | -1.92 (-4.91, 1.07) | p=0.65 | Urine epinephrine-to-creatinine ratio difference | -3.14 (-6.58, 0.30) | p=0.11 |
| Coughlin 2007 | NM | NM |  | BRS | 1. (-0.1, 2.2)   (adequate compliance 1.4 (0.1, 2.7) ) | 0.07 (adequate compliance <0.04) |
| Craig 2012 | NM | NM |  | NM | NM |  |
| Drager 2007 | CRP | CPAP group 3.7±1.8 to 2.0±1.2* | p=0.001 | Catecholamine | CPAP group 365±125 to 205±51* | p <0.001 |
|  |  | Control group 3.1±2.8 to 3.3±2.7* | NS |  | Control group 362±151 to 357±106* | NS |
| Phillips 2011 | NM | NM |  | Urine adrenaline | -0.6 (-7.6, 6.4) | p=0.87 |
|  |  |  |  | Urine noradrenaline | -67.1 (-117.7, -16.4) | p=0.01 |
| Robinson 2004 | Homocysteine | -0.18 (-1.87, 1.51) | p=0.83 | NM | NM |  |
| Sharma 2011 | NM | NM |  | NM | NM |  |

**Footnotes**

Abbreviations: NM, not mentioned; CRP, C-reactive protein; BRS, baroreceptor sensitivity; NS, not significant

***** expressed as mean ± SD

**Table S2B. Subgroup analysis by inflammatory mediators or autonomic activity marker effect for CPAP effects on lipid profiles**

|  | **Origin main results: mean (95%CI), mg/dl** | **Subgroup by inflammatory mediators effect: mean (95%CI), mg/dl** | **Subgroup by autonomic activity marker effect: mean (95%CI), mg/dl** |
| --- | --- | --- | --- |
| **T-CHO** | -6.23 (-8.73,-3.73) | Unchanged: -3.55 (-8.16, 1.05)  Reduced effect: -5.0 (-34.96, 24.96) * | Unchanged: -2.61 (-7.64, 2.41)  Reduced effect: -7.32 (-10.40, -4.25) |
| **TG** | -12.60 (-18.80,-6.41) | Unchanged: -5.31 (-15.05, 4.44)  Reduced effect: 19.0 (-37.42, 75.42) * | Unchanged: -4.82 (-14.42, 4.78)  Reduced effect: -18.22 (-26.54, -9.89) |
| **HDL** | -1.05 (-1.69,-0.40) | Unchanged: -0.69 (-2.00, 0.63)  Reduced effect: -1.00 (-8.09, 6.09) * | Unchanged: -0.78 (-1.96, 0.41)  Reduced effect: -1.16 (-1.93, -0.39) |
| **LDL** | -1.01 (-5.04,3.02) | Unchanged: -0.32 (-4.56, 5.20)  Reduced effect: -4.0 (-29.88, 21.88)* | Unchanged: -0.93 (-5.02, 3.148)  Reduced effect: -4.0 (-29.88, 21.88)* |

**Footnotes**

*Only one study included

**Supplement S3.** Analysis for publication bias. FE, possible missing studies filled by *trim and fill* as a guide; vertical black dotted line: the pooled effect estimate on the original meta-analysis; vertical gray dotted line: the pooled estimate, including the filled studies. Contour-enhanced funnel plot with *trim and fill* for primary outcome of **(S3A)** total cholesterol (*p*=0.348, Begg’s test; *p*=0.428, Egger’s test), **(S3B)** triglyceride (*p*=0.851, Begg’s test; p=0.756, Egger’s test), **(S3C)** high-density lipoprotein (*p*=0.624, Begg’s test; *p*=0.794, Egger’s test), and **(S3D)** low-density lipoprotein (*p*=0.497, Begg’s test; *p*=0.953, Egger’s test).

**Figure S3A**

**
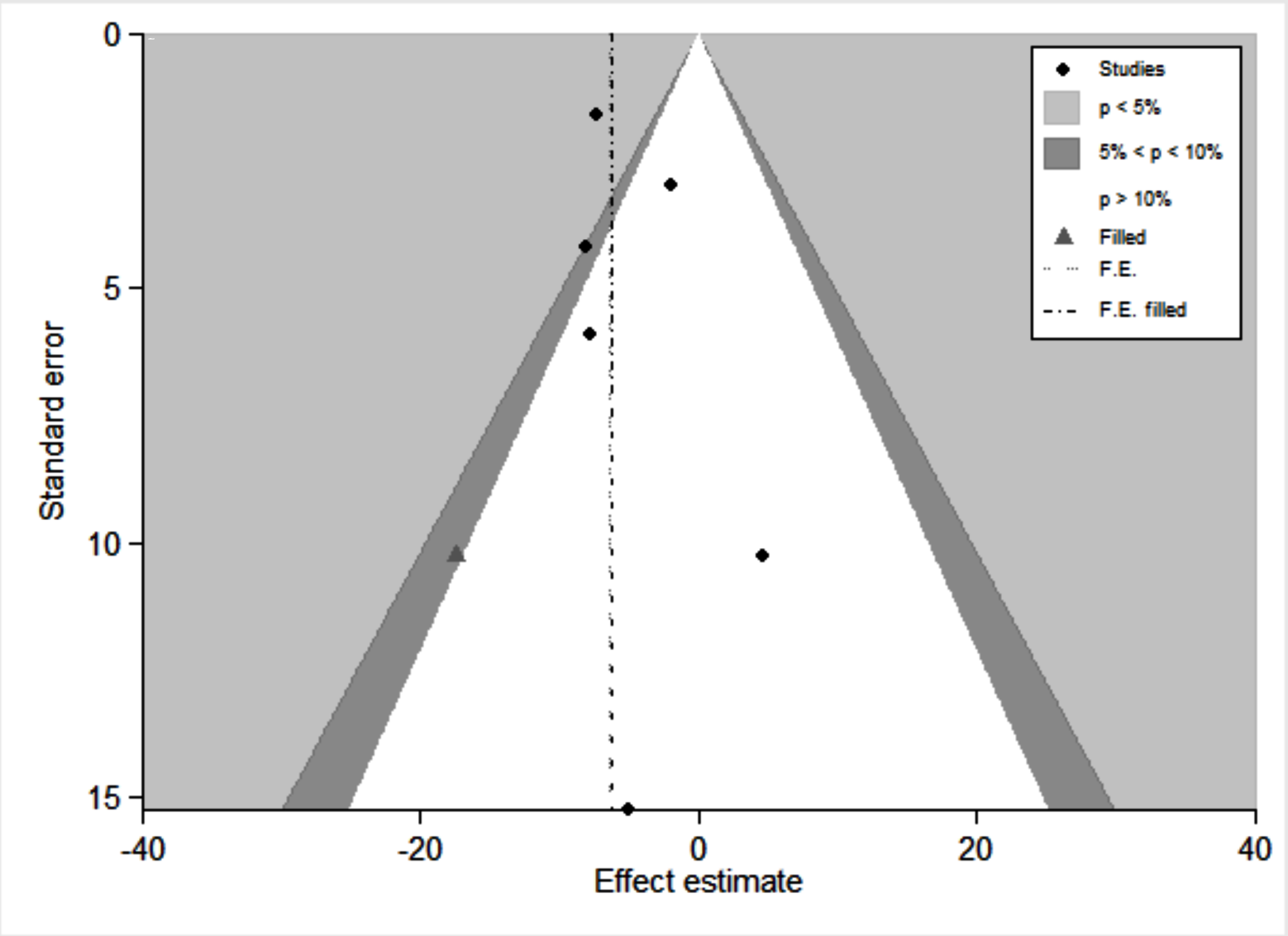
**

**Figure S3B**

**
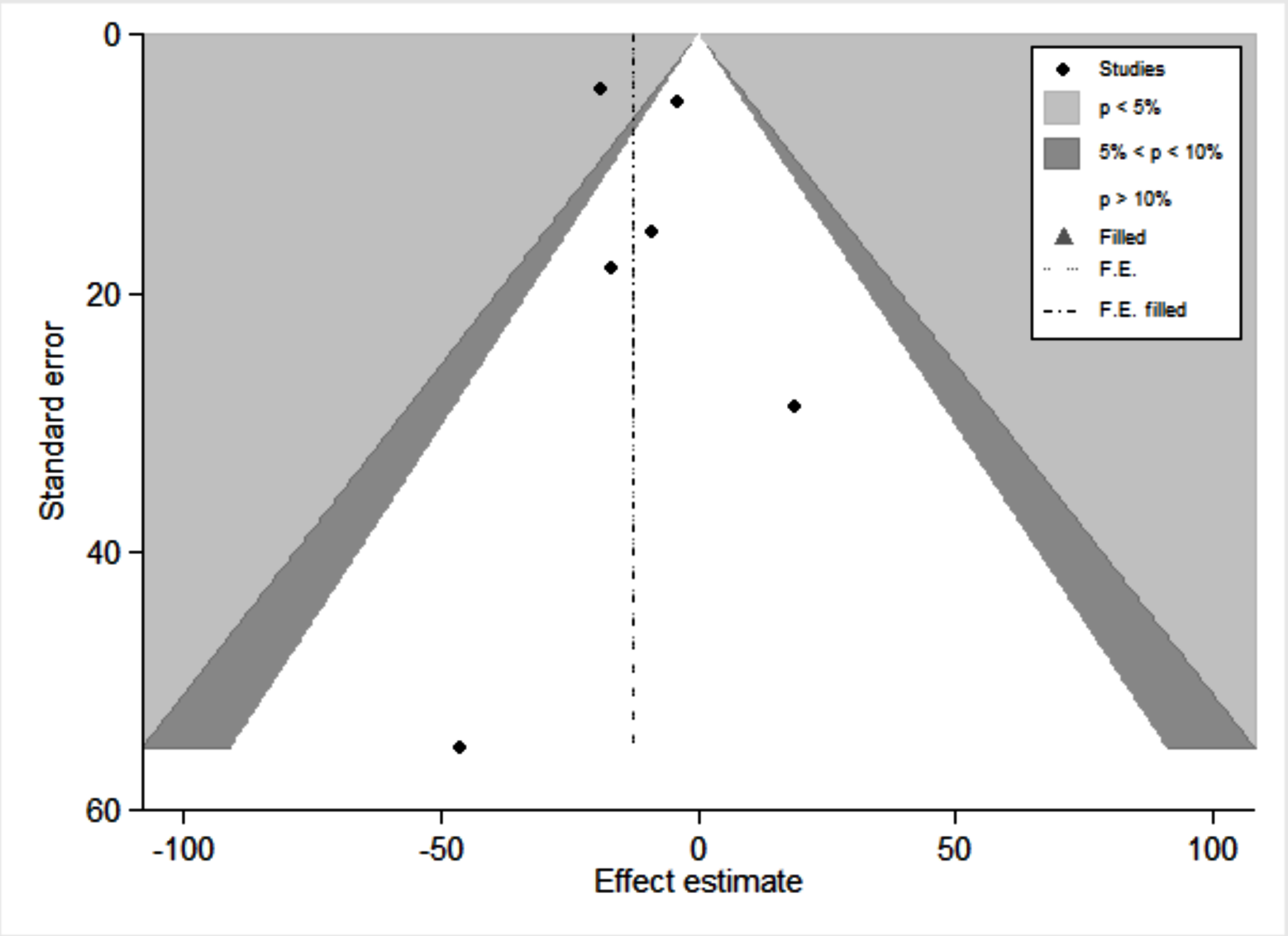
**

**Figure S3C**

**
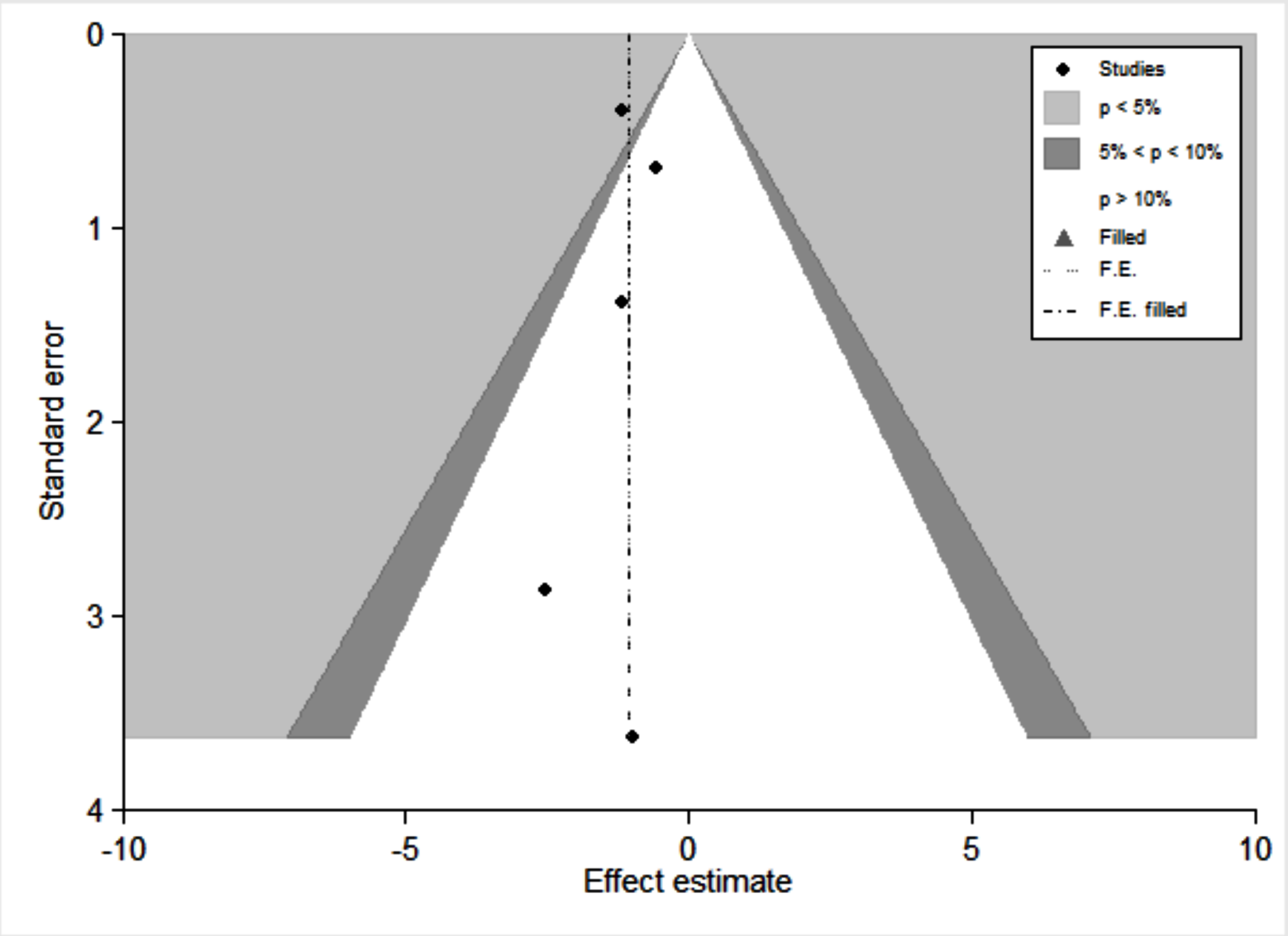
**

**Figure S3D**

**
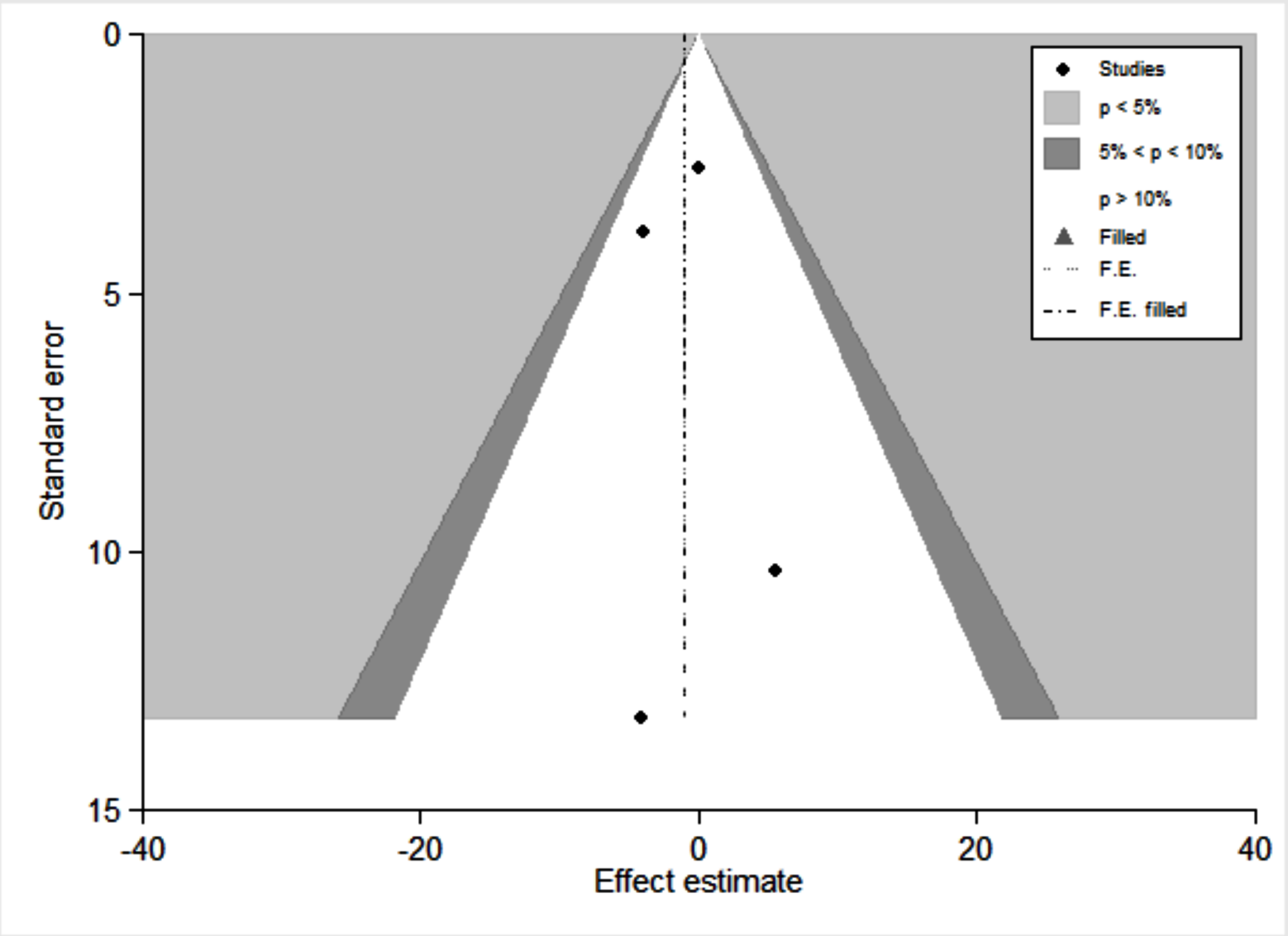
**

**Supplement S4.** Sensitivity analysis for total cholesterol (T-CHO), triglyceride (TG), high-density lipoprotein (HDL), and low-density lipoprotein (LDL) by adjusting Drager's Study [[4](#_ENREF_4)], excluding high risk of bias and body weight change, and including Sharma’s study [[7](#_ENREF_7)].

Drager's study [[4](#_ENREF_4)] reported only before-after results of lipid profiles under CPAP treatment without providing correlation in between. For this kind of paired data, correlation was needed to calculate for standard deviation of mean differences. Sensitivity analysis was conducted for T-CHO, TG, HDL, and LDL by adjusting the correlation of Drager's study. Here, three correlation conditions (0.2, 0.5, and 0.8, with 0.5 as default) were set for all of the four factors to investigate I^2^ and the main effect in both fixed-effect and random-effects models if there was obvious heterogeneity (Table S4A). By tuning the correlation, the I^2^ of three factors did not dramatically change. While testing the main effect of each factor under different correlation condition, the direction of main effects on all lipid profiles was the same; the magnitude of main effects was relatively consistent. As for sub-group analysis and meta-regression, the results were similar (Table S4A).

**S4A.** Sensitivity analysis based on studies with before-after correlation

|  | **Correlation** | **I^2^** | **Main results (fixed-effect)** | **Subgroup analysis (fixed-effect)** | **Meta-regression (fixed-effect)** |
| --- | --- | --- | --- | --- | --- |
| **T-CHO** | γ=0.5 (default) | 0% | -6.23 (-8.73,-3.73)* |  |  |
|  | γ=0.2 | 0% | -6.24 (-8.74,-3.73)* |  |  |
|  | γ=0.8 | 0% | -6.22 (-8.71,-3.73)* |  |  |
| **TG** | γ=0.5 (default) | 25% | -12.60 (-18.80,-6.41)* | Study design (p=0.027)  Control type (p=0.025)  OSA severity (p=0.037)  Daytime sleepiness (p=0.044)  CPAP duration (p=0.020)  CPAP compliance (p=0.031)  Risk of bias (p=0.033) | CPAP duration (β=0.87, p=0.029)  HTN (β=0.23, p=0.049)  CVD (β=2.30, p=0.040) |
|  | γ=0.2 | 20.3% | -12.74 (-18.94,-6.53)* | Study design (p=0.030)  Control type (p=0.028)  OSA severity (p=0.034)  Daytime sleepiness (p=0.041)  CPAP duration (p=0.023)  CPAP compliance (p=0.029)  Risk of bias (p=0.036) | Age (β=1.49, p=0.046)  CPAP duration (β=0.87, p=0.030)  CPAP compliance (β=-7.32, p=0.047)  HTN (β=0.24, p=0.040)  CVD (β=2.34, p=0.036) |
|  | γ=0.8 | 37.6% | -12.19 (-18.34,-6.04)* | Study design (p=0.019)  Control type (p=0.017)  OSA severity (p=0.048)  CPAP duration (p=0.014)  CPAP compliance (p=0.042)  Risk of bias (p=0.023) | CPAP duration (β=0.89, p=0.026)  CPAP compliance (β=-7.32, p=0.047) |
| **HDL** | γ=0.5 (default) | 0% | -1.05 (-1.69,-0.40)* |  |  |
|  | γ=0.2 | 0% | -1.05 (-1.69,-0.40)* |  |  |
|  | γ=0.8 | 0% | -1.04 (-1.69,-0.40)* |  |  |
| **LDL** | γ=0.5 (default) | 0% | -1.01 (-5.04,3.02) |  |  |
|  | γ=0.2 | 0% | -0.98 (-5.03,3.07) |  |  |
|  | γ=0.8 | 0% | -1.10 (-5.07,2.86) |  |  |

**Footnotes:** γ, correlation

**p*<0.05

When excluding studies with high risk of bias, including performance bias in Comondore’s, Craig’s, and Drager’s studies [[1](#_ENREF_1),[3](#_ENREF_3),[4](#_ENREF_4)] and attrition bias in Craig’s and Robinson’s studies [[3](#_ENREF_3),[6](#_ENREF_6)], there were no change in the direction of the main results in all outcomes, while the magnitude of main effects became more prominent (Table S4B). Body weight changes was also used as surrogate for possible confounders of diet, physical activity, or body composition. Excluding studies [[1](#_ENREF_1),[3](#_ENREF_3),[6](#_ENREF_6)] that had reported changes in body weight from pooled analyses, the magnitude and direction of the effects on all lipid-lowering effects did not change (Table S4B).

**S4B.** Sensitivity analysis based on risk of bias or on body weight change or including Sharma’s study

|  | **Origin main results: mean (95%CI), mg/dl** | **Results excluding studies of high risk of bias*: mean (95%CI), mg/dl** | **Results excluding studies of unclear or reported change of body weight^#^: mean (95%CI), mg/dl** | **Results including Sharma’s study (7) : mean (95%CI), mg/dl** |
| --- | --- | --- | --- | --- |
| **T-CHO** | -6.23 (-8.73,-3.73) | -7.37 (-10.36,-4.38) | -7.35 (-10.32,-4.38) | -6.86 (-9.24,-4.47) |
| **TG** | -12.60 (-18.80,-6.41) | -18.29 (-26.39, -10.19) | -17.54 (-25.56,-9.52) | -13.02 (-18.99,-7.04) |
| **HDL** | -1.05 (-1.69,-0.40) | -1.16 (-1.90,-0.42) | -1.16 (-1.90, -0.42) | -1.02 (-1.65,-0.38) |
| **LDL** | -1.01 (-5.04,3.02) | -3.87 (-11.32,3.58) | -3.88 (-11.04, 3.28) | -3.10 (-6.60,0.41) |

**Footnotes**

*Excluding studies of [Comondore 2009](#STD-Comondore-2009), Craig 2012, [Drager 2007](#STD-Drager-2007), and [Robinson 2004](#STD-Robinson-2004)

^#^Excluding studies of [Comondore 2009](#STD-Comondore-2009), Craig 2012, and [Robinson 2004](#STD-Robinson-2004)

**Supplement S5.** GRADE evidence profiles for continuous positive airway pressure (CPAP) effects on lipid metabolism in patients with obstructive sleep apnoea (OSA)

| **Quality assessment** | | | | | | | **Summary of Findings** | | | | |
| --- | --- | --- | --- | --- | --- | --- | --- | --- | --- | --- | --- |
| **Participants (studies) Follow up** | **Risk of bias** | **Inconsistency** | **Indirectness** | **Imprecision** | **Publication bias** | **Overall quality of evidence** | **Study event rates (%)** | | **Relative effect** (95% CI) | **Anticipated absolute effects** *Time frame is 10.6 wks (2~24wks)* | |
|  |  |  |  |  |  |  | **With Control** | **With CPAP** |  | **Risk with Control** | **Risk difference with CPAP** (95% CI) |
| **total cholesterol** (CRITICAL OUTCOME; measured with: biochemical assay; Better indicated by lower values) | | | | | | | | | | | |
| 699 (6 studies) 10.3 weeks | no serious risk of bias | no serious inconsistency | no serious indirectness | no serious imprecision | undetected | ⊕⊕⊕⊕ **HIGH** | 351 | 348 | **-** | The mean total cholesterol ranged across control groups from  **-2.71 to 0.77 mg/dl**^1^ | The mean total cholesterol in the intervention groups was **6.23 lower** (8.73 to 3.73 lower) |
| **triglyceride** (CRITICAL OUTCOME; measured with: biochemical assay; Better indicated by lower values) | | | | | | | | | | | |
| 609 (6 studies) 10.3 weeks | no serious risk of bias | no serious inconsistency | no serious indirectness^2^ | no serious imprecision | undetected | ⊕⊕⊕⊕ **HIGH**^2^ | 303 | 306 | **-** | The mean triglyceride ranged across control groups from  **-7 to 32.77 mg/dl**^1^ | The mean triglyceride in the intervention groups was **12.60 lower** (18.80 to 6.41 lower) |
| **high-density lipoprotein** (CRITICAL OUTCOME; measured with: biochemical assay; Better indicated by higher values) | | | | | | | | | | | |
| 512 (5 studies) 11.6 weeks | no serious risk of bias | no serious inconsistency | no serious indirectness | no serious imprecision | undetected | ⊕⊕⊕⊕ **HIGH** | 258 | 254 | **-** | The mean high-density lipoprotein ranged across control groups from  **-1 to 1.55 mg/dl**^3^ | The mean high-density lipoprotein in the intervention groups was **1.05 lower** (1.69 to 0.40 lower) |
| **low-density lipoprotein** (CRITICAL OUTCOME; measured with: biochemical assay; Better indicated by lower values) | | | | | | | | | | | |
| 450 (4 studies) 12.5 weeks | no serious risk of bias | no serious inconsistency | no serious indirectness^2^ | no serious imprecision | undetected | ⊕⊕⊕⊕ **HIGH**^2^ | 225 | 225 | **-** | The mean low-density lipoprotein ranged across control groups from  **-2.32 to-2 mg/dl**^3^ | The mean low-density lipoprotein in the intervention groups was **1.01 lower** (5.04 lower to 3.02 higher) |

^1^The study by Phillips and Coughlin did not report before-after change of the TG level in the control group.
^2^Non-fasting blood sampling was used in Robinson and Phillips study. Drager’s study only reported blood sampling between 8:00 and 10:00 A.M. However, other data all reported fasting TG levels.
^3^The study by Robinson, Phillips, and Coughlin did not report before-after change of HDL and LDL levels in control group.**REFERENCES FOR SUPPLEMENT**

1. Comondore VR, Cheema R, Fox J, Butt A, John Mancini GB, Fleetham JA, Ryan CF, Chan S, Ayas NT (2009) The impact of CPAP on cardiovascular biomarkers in minimally symptomatic patients with obstructive sleep apnea: a pilot feasibility randomized crossover trial. Lung 187 (1):17-22. doi:10.1007/s00408-008-9115-5

2. Coughlin SR, Mawdsley L, Mugarza JA, Wilding JP, Calverley PM (2007) Cardiovascular and metabolic effects of CPAP in obese males with OSA. Eur Respir J 29 (4):720-727. doi:10.1183/09031936.00043306

3. Craig SE, Kohler M, Nicoll D, Bratton DJ, Nunn A, Davies R, Stradling J (2012) Continuous positive airway pressure improves sleepiness but not calculated vascular risk in patients with minimally symptomatic obstructive sleep apnoea: the MOSAIC randomised controlled trial. Thorax 67 (12):1090-1096. doi:10.1136/thoraxjnl-2012-202178

4. Drager LF, Bortolotto LA, Figueiredo AC, Krieger EM, Lorenzi GF (2007) Effects of continuous positive airway pressure on early signs of atherosclerosis in obstructive sleep apnea. Am J Respir Crit Care Med 176 (7):706-712. doi:10.1164/rccm.200703-500OC

5. Phillips CL, Yee BJ, Marshall NS, Liu PY, Sullivan DR, Grunstein RR (2011) Continuous positive airway pressure reduces postprandial lipidemia in obstructive sleep apnea: a randomized, placebo-controlled crossover trial. Am J Respir Crit Care Med 184 (3):355-361

6. Robinson GV, Pepperell JC, Segal HC, Davies RJ, Stradling JR (2004) Circulating cardiovascular risk factors in obstructive sleep apnoea: data from randomised controlled trials. Thorax 59 (9):777-782. doi:10.1136/thx.2003.018739

7. Sharma SK, Agrawal S, Damodaran D, Sreenivas V, Kadhiravan T, Lakshmy R, Jagia P, Kumar A (2011) CPAP for the metabolic syndrome in patients with obstructive sleep apnea. N Engl J Med 365 (24):2277-2286. doi:10.1056/NEJMoa1103944
